# Association between serum uric acid levels and cardiovascular risk among university workers from the State of Mexico: a nested case–control study

**DOI:** 10.1186/1471-2458-13-415

**Published:** 2013-05-01

**Authors:** Patricia Cerecero, Bernardo Hernández-Prado, Edgar Denova, Roxana Valdés, Gilberto Vázquez, Eneida Camarillo, Gerardo Huitrón

**Affiliations:** 1 Centro de Investigación en Ciencias Médicas, Universidad Autónoma del Estado de México, Toluca, México; 2 Institute for Health Metrics and Evaluation, University of Washington, Seattle, WA, USA; 3 Instituto Nacional de Salud Pública, Cuernavaca, Morelos, México; 4 Centro de Investigación y Estudios de Posgrado en Ciencias de la Salud Universidad Autónoma del Estado de México, Toluca, México

**Keywords:** Uric acid, Cardiovascular diseases, Risk factors, Mexico

## Abstract

**Background:**

Recent evidence suggests that serum uric acid (SUA) can be an inexpensive and easy-to-obtain indicator of cardiovascular risk (CR). This is especially important in developing countries with high prevalence of cardiovascular disease. We examined the association between SUA levels and 10-year global CR among university workers from the State of Mexico, Mexico.

**Methods:**

A case–control study nested within a cohort was conducted between 2004 and 2006. Anthropometric measures, lifestyle variables, family background and CR factors were assessed. The analysis estimated odds ratios using conditional logistic regression.

**Results:**

The study included 319 cases with CR and 638 controls. Subjects in the upper tertile of SUA had 48.0% higher odds of having an elevated CR than those in the lower tertile (OR = 1.48, 95% CI: 1.04 - 2.10) in the crude analysis, but the association was non-significant when adjusting for other covariates. Among physically inactive individuals, being in the third tertile of SUA doubled the odds of high CR, compared with those who perform physical activity three or more hours per week being in the first tertile of SUA (OR = 2.35, 95% CI: 1.24 - 4.45).

**Conclusion:**

Serum concentration of uric acid is associated with 10-year global CR among individuals with high levels of physical inactivity.

## Background

Recent studies show that concentrations of serum uric acid (SUA) can be an inexpensive and easy-to-obtain indicator of cardiovascular risk (CR) [1,2]. This is especially relevant in Mexico, where the prevalence of cardiovascular disease has increased alarmingly during the past two decades [3]. The epidemiological and nutritional transition the country is undergoing has resulted in a high prevalence of CR factors in the adult population (e.g. prevalence of 70% for overweight and obesity, 26.5% for hypertension or hypercholesterolemia, and 13.5% for diabetes mellitus [4].

The global CR is determined based on the degree of exposure to a set of risk factors for cardiovascular disease. It expresses the probability of an individual to develop an adverse cardiovascular event during a specific period of time in comparison with a same-aged and same-gender individual without risk factors [5].

Uric acid is the end-product of purine metabolism. It comes from the conversion of hypoxanthine into xanthine and from xanthine into uric acid; both reactions are catalyzed by the enzyme xanthine oxidoreductase [6]. SUA levels over 7 mg/dL in men and over 6 mg/dL in women are considered high, although the parameter can vary considerably according to the geographical area and the ethnic group [7]. It has been shown elevated uric acid has a harmful effect on platelets and on endothelial function [8]. It has also been demonstrated that reducing its concentration through the use of drugs such as atorvastatin [9] or allopurinol [10] is associated with a reduced prevalence of cardiovascular disease. A growing body of evidence has demonstrated that elevated SUA levels are associated with many of the risk factors for cardiovascular diseases (such as hypertension [11], obesity and hypertriglyceridemia [12]); with some of the factors that characterize atherosclerosis (such as inflammation, oxidative stress and endothelial dysfunction [8,10]); and with lifestyle factors (i.e., physical inactivity, inadequate dietary habits and elevated alcohol intake [13,14]). In Mexico, there is no evidence of the precise role that uric acid plays in the risk of developing a cardiovascular disease. Hence, the main purpose of our study was to determine whether high levels of uric acid are a marker associated with 10-year CR in university workers from the State of Mexico.

## Methods

### Population and sample

Data for this analysis come from a cohort study with workers of the Authonomous University of the State of Mexico (UAEMex, for its name in Spanish), named the “Cohort of UAEMex Workers”. This study has been conducted since 2004 jointly by the Mexican Institute of Social Security and the National Institute of Public Health of Mexico. The detailed description of the methodology has been published elsewhere [15,16].

The baseline measurement of the cohort study included 2,555 university workers. In the present analysis we included the data of 2,065 participants over 30 years old who provided complete information and were free of diagnosed cardiovascular disease. The data of 45 participants with gout diagnosis, diuretic treatment or kidney failure (serum creatinine ≥1.5 mg/dL) were excluded. We also excluded the data of 317 subjects who reported implausible total energy intake (<500 or >7000 kcal/day).

### Study design

**Figure 1:**
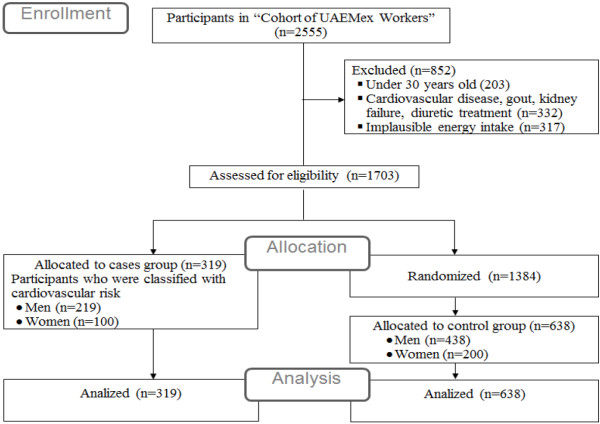
Flow diagram of eligibility and allocation of participants in “Cohort of UAEMex Workers”.

We conducted a case–control study nested in the cohort mentioned above. To define the cases, the 10-year CR for each participant was calculated through the method proposed by Wilson *et al.*[5]. It proposes a risk scoring method considering the degree of exposure to the following variables: age, total cholesterol, high-density lipoproteins cholesterol (HDL-chol), blood pressure, diabetes mellitus and smoking status. In accordance with the ILIB-Latin America [17] consensus, the CR is classified as latent (< 10.0%), intermediate (≥ 10.0% and < 20%) or high (≥ 20.0%). Therefore, a case was defined as any participant from the cohort with intermediate or high CR. Controls were defined as subjects with latent CR, and were matched with case subjects by gender, selecting randomly two controls per case. In total 319 cases were classified (Figure 1).

### Data collection

We visited all schools that are part of the UAEMex to invite workers to participate in the study. The survey, applied between 2004 and 2006, included a questionnaire, a physical examination and analyses of blood samples. After written informed consent was obtained, participants were given an auto-administered questionnaire to be filled at home. The questionnaire collected data on gender, lifestyle (diet, tobacco and alcohol consumption, and physical activity,) as well as family history of cardiovascular disease. It was to be delivered within eight days.

The data on diet and alcohol consumption were obtained using a semi-quantitative food frequency questionnaire validated for the Mexican population [18]. Questions were asked on the frequency of consumption of a standard portion of 116 types of foods with ten possible answers (ranged from “never” to “six or more a day”) and eight different types of beverages with eleven possible answers (ranged from “zero” to “more than 15 glasses”), during the past year. The diet components analyzed included: the daily intake of total energy, fructose, lipids and proteins.

The level of leisure-time physical activity was assessed through a questionnaire designed to estimate the intensity and duration of different types of activities in a typical week during the past year, taking into account only those that generate an energetic expenditure ≥ 3 metabolic equivalents (MET) (walking, running, bicycling, playing soccer, etc.) [19], with seven possible answers (ranged from “5 minutes” to “more than 6 hours a week”). Workers were classified according to the time they spend in physical activity following the Pan American Health Organization recommendations [20]: recommended activity (≥ 3 hours/week), insufficient activity (< 3 hours/week) and inactivity (0 hours/week).

Once the subjects handed in the answered questionnaire, they had an appointment at a laboratory in the Medical Sciences Research Center (*Centro de Investigación en Ciencias Médicas* – CICMED-UAEMex) to conduct blood sampling, height, weight and blood pressure measurements. All measurement procedures were performed by nurses trained to use standardized procedures [21]. Weight and height were measured after overnight fasting using an electronic scale (Tanita model BC-533; Tokyo, Japan) and a conventional stadiometer with participants wearing minimal clothing and no shoes. Blood pressure was measured with an automatic digital monitor (model CH 656C). Three assessments were carried out at two-minute intervals and the average was registered.

Blood samples were collected after an overnight fasting of at least 12 hours, without having performed intense physical activity or having ingested alcohol, following standarized procedures; the samples were immediately centrifuged and processed with an automatic device. Serum levels of total cholesterol, high density lipoproteins cholesterol (HDL-chol), glucose, and uric acid were determined enzymatically on a standard autoanalizer (Selectra XL, Randox). The Research Ethics Committee at CICMED-UAEMex approved the procedures used and all subjects signed an informed consent letter before data collection.

### Analysis

For the analysis, body mass index (BMI) was calculated by dividing the body weight (in kilograms) by the square height (in meters). With this index, workers were grouped into normal weight (BMI ≤ 24.9) and overweight-obesity (BMI ≥ 25) [22]. SUA values were classified according to their distribution in tertiles. The CR factors were defined as follows [5]: age, men ≥ 40 years, women ≥ 45 years; high cholesterol, ≥ 200 mg/dl; low HDL-chol, men ≤ 45 mg/dl, women ≤ 50 mg/dl; diabetes, fasting serum glucose ≥ 110 mg/dl and/or treatment for diabetes; systolic/diastolic hypertension, men ≥ 140/90 mmHg, women ≥ 130/85 mmHg; and smoking status, yes or no.

We performed a descriptive analysis of workers characteristics. Means and standard deviations (SD) for continuous variables and proportions for categorical variables for the cases and controls were compared by linear regression adjusted for matching and the clustering of observations at school level and chi-square test. We computed the prevalence for lifestyle variables, family history of cardiovascular disease and factors that constitute the CR (age, hypertension, elevated cholesterol, low HDL-chol, diabetes, and smoking) by tertiles of SUA.

To estimate the magnitude of the association between categories of SUA and high CR (10-year risk more than 10 percent), as well as between SUA and each of CR factors, we computed crude and adjusted OR and 95% confidence intervals (95% CI) using conditional logistic regression. Furthermore, we evaluated the interaction of physical activity level and uric acid concentrations on CR by introducing an interaction term in the logistic regression models.

The Mantel–Haenszel extension test was used to assess linear trend of the OR for high CR, each of the CR factors, and BMI >25 kg/m^2^ across increasing tertiles of uric acid. The nutrient intake was adjusted for total energy intake using the residual method [23].The differences with a ρ-value <0.05 were considered significant. All analyses were performed using STATA statistical software, version 7 [24].

## Results

The study sample was constituted by 957 university workers, 319 of which were CR cases (244 in intermediate CR, 75 in high CR) and 638 controls; 31.4% were female and 68.6% male. The mean age was 45.3 years (SD = 8.4). Mean SUA concentration was 4.8 mg/dL (SD = 1.3 mg/dl) and mean probability of having a cardiovascular adverse event in the next ten years was 8.3%. Most workers were overweight or obese (72.0%) and declared consuming at least one alcoholic beverage a day (84.2); 36.0% reported being physically inactive. Out of the factors that constitute global CR, 75.6% of the sample displayed low HDL-chol values and nearly half displayed high cholesterol or hypertension (Table 1).

**Table 1:** Clinical and anthropometric characteristics and lifestyle conditions of university workers in the State of Mexico, Mexico, 2004

**Characteristics**	**Mean**	**SD**
Age, y	45.3	8.4
Serum uric acid, mg/dL	4.8	1.3
Fructose intake, g/day ^a^	30.4	16.0
Lipids intake, g/day ^a^	61.0	16.0
Proteins intake, g/day ^a^	80.4	17.7
	**n**	**%**
Body mass index, kg/m^2^		
< 25 (normal weight)	269	28.0
≥ 25 (overweight-obesity)	688	72.0
Alcoholic beverages consumption		
0 glasses/day	118	12.3
< 2 glasses/day	806	84.2
≥ 2 glasses/day	33	3.5
Physical activity (hours/week spent in activities ≥ 3 MET)		
Inactivity (0 hours)	344	36.0
Insufficient activity (< 3 hours)	294	30.7
Recommended (≥ 3 hours)	319	33.3
Health family history		
Myocardial infarction	207	21.6
Type 2 diabetes	484	50.5
Hypertension	493	51.5
Cardiovascular risk factors		
Age, y (≥ 40 men, ≥ 45 women)	587	61.3
Hypertension	446	46.6
High cholesterol level (> 200 mg/dL)	459	47.9
Low HDL-chol level (< 45 mg/dL men, < 50 mg/dL women)	724	75.6
Type 2 diabetes	151	15.7
Smoking	167	17.4
Number of cardiovascular risk factors		
0 - 2	448	46.8
3 – 6	509	53.2

^a^ Consumption estimates of fructose, lipids and proteins were energy adjusted using the residual method [23].

The proportion of overweight workers among the cases surpassed that of the controls (ρ < 0.001). Likewise, fructose intake was higher among the cases with respect to the controls (ρ = 0.028) (Table 2).

**Table 2:** Comparison of clinical and anthropometric characteristics and lifestyle conditions among university workers with and without cardiovascular risk, 2004

**Characteristics**	**% Cases (with high CR) n = 319**	**% Controls n = 638**	***p *value **^**a**^
Tertiles of serum uric acid			
1 (≤ 4.18 mg/dL)	29.7	35.1	0.099
2 (4.19 - 5.34 mg/dL)	33.5	33.4	0.961
3 (≥ 5.35 mg/dL)	36.8	31.5	0.109
Body mass index, kg/m^2^			
< 25 (normal weight)	19.2	32.6	<0.001
≥ 25 (overweight-obesity)	80.8	67.4	
Alcoholic beverages consumption			
0 glasses/day	12.2	12.4	0.754
< 2 glasses/day	83.7	84.5	
≥ 2 glasses/day	4.1	3.1	
Physical activity (hours/week spent in activities >3 MET)			
Inactivity (0 hours)	40.1	33.8	0.057
Insufficient activity (< 3 hours)	31.1	30.6	0.882
Recommended (≥ 3 hours)	28.8	35.6	0.037
Health family history			
Myocardial infarction	24.7	20.0	0.096
Type 2 diabetes	50.1	50.8	0.855
Hypertension	54.8	49.8	0.143
Cardiovascular risk factors:			
Age, y (≥40 men, ≥45 women)	94.3	44.8	<0.001
Hypertension	74.6	32.6	<0.001
High total cholesterol	64.3	39.8	<0.001
Low HDL-chol	83.1	72.0	<0.001
Type 2 diabetes	38.0	4.7	<0.001
Smoking	23.8	14.2	<0.001
Daily intake, g	**Mean (SD)**	**Mean (SD)**	
Fructose ^b^	32.4 (17.0)	29.3 (15.5)	0.028
Lipids ^b^	59.9 (15.0)	61.6 (16.4)	0.091
Proteins ^b^	80.8 (16.6)	80.2 (18.3)	0.693

^a^*p* value for categorical variables refer to the comparison of cases vs. controls using chi-square tests, and for continuous variables refer to the linear regression analysis adjusted for the effect of matching and clustering at the school level.

^b^ Intake estimates of fructose, lipids and proteins were energy adjusted using the residual method [23].

CR: cardiovascular risk.

Table 3 shows that subjects in highest tertile of SUA distribution tended to be more physically active, consume more alcoholic beverages per day, have hypertension, have high total cholesterol, have low HDL-chol, being overweight or obese, and have more than three cardiovascular risk factors compared with subjects in the lowest tertile of SUA. In addition, subjects with higher uric acid levels were older than those with lower levels.

**Table 3:** Lifestyle conditions and cardiovascular risk factors according to tertiles of serum uric acid in university workers of State of Mexico, 2004

	**Tertiles of serum uric acid **^**b**^			
	**T1**	**T2**	**T3**	***p *value **^**a**^
	**(n = 319)**	**(n = 320)**	**(n = 318)**	
BMI ≥25 kg/m^2^,%	60.8	74.0	80.8	<0.001
Alcoholic beverages consumption,%				
0 glasses/day	16.5	11.0	9.4	0.011
< 2 glasses/day	81.0	86.5	85.2	
≥ 2 glasses/day	2.5	2.5	5.3	
Physical activity (hours/week spent in activities >3 MET),%				
Inactivity (0 hours)	44.8	31.5	31.4	0.001
Insufficient activity (< 3 hours)	28.5	32.5	31.1	
Recommended (≥ 3 hours)	26.7	36.0	37.5	
Health family history,%				
Myocardial infarction	36.7	35.3	28.0	0.189
Type 2 diabetes	31.0	34.0	35.0	0.276
Hypertension	36.5	31.7	31.8	0.098
Daily intake (g), mean				
Fructose	30.7	29.8	30.6	0.830
Lipids	59.6	62.3	61.1	0.268
Proteins	79.9	80.2	81.1	0.806
Cardiovascular risk factors,%				
Age, y (≥40 men, ≥45 women)	57.9	67.8	58.2	0.014
Hypertension	38.0	47.5	54.4	<0.001
High total cholesterol	43.0	45.6	55.4	0.004
Low HDL-chol	70.5	75.6	80.8	0.010
Type 2 diabetes	15.4	85.0	17.0	0.766
Smoking	17.5	16.6	18.2	0.854
≥3 cardiovascular risk factors,%	46.0	54.0	59.4	0.003

^a^*p* value for categorical variables refer to the comparison between tertiles of SUA using chi-square tests, and for continuous variables refer to the linear regression analysis adjusted for the effect of matching and clustering at the school level.

^b^ Tertile 3: ≥ 5.35 mg/dL; tertile 2: 4.19 - 5.34 mg/dL; tertile 1: ≤ 4.18 mg/dL.

The odds of presenting high 10-year CR estimated as well as of presenting each of its components (age, hypertension, elevated cholesterol, low HDL-chol, diabetes, and smoking), and overweight-obesity (BMI ≥25 kg/m^2^) according to SUA levels are show in Table 4. The crude analysis showed that subjects in higher tertile of SUA have 40.0% greater odds of presenting high 10-year CR, compared to those in lower tertile (OR = 1.41, 95% CI: 1.01-1.98). This association remained with little change after controlling for the effect of physical activity, alcoholic beverages consumption, and family history of myocardial infarction (OR = 1.48, 95% CI: 1.04-2.10). However, after adjusting for BMI in addition to the above mentioned variables, the magnitude of the relationship between SUA levels and high cardiovascular risk decreased and lost statistical significance (OR = 1.28, 95% CI: 0.89-1.83).

**Table 4:** Odds ratio of cardiovascular risk or its components according to tertiles of uric acid in university workers of State of Mexico, 2004

**Dependent variables**	**Uric acid levels **^**c**^	**Crude odds ratio (95% CI)**	**Multivariate-adjusted **^**a **^**odds ratio (95% CI)**	**Multivariate-adjusted **^**b **^**odds ratio (95% CI)**
High cardiovascular risk	Tertile 3	1.41 (1.01, 1.98)	1.48 (1.04, 2.10)	1.28 (0.89, 1.83)
(10-year CR ≥10.0%)	Tertile 2	1.22 (0.87, 1.72)	1.28 (0.90, 1.81)	1.19 (0.83, 1.69)
	Tertile 1	1	1	1
	Trend over tertiles, p	0.060	0.031	0.122
Cardiovascular risk factors:				
Age, y (≥ 40 men, ≥ 45 women)				
	Tertile 3	1.27 (0.87, 1.85)	1.28 (0.87, 1.87)	1.15 (0.78, 1.71)
	Tertile 2	1.51 (1.04, 2.18)	1.53 (1.04, 2.24)	1.47 (1.00, 2.17)
	Tertile 1	1	1	1
	Trend over tertiles, p	0.960	0.805	0.737
Hypertension	Tertile 3	1.93 (1.32, 2.82)	1.86 (1.26, 2.74)	1.65 (1.11, 2.45)
	Tertile 2	1.48 (1.01, 2.17)	1.43 (0.96, 2.12)	1.36 (0.91, 2.04)
	Tertile 1	1	1	1
	Trend over tertiles, p	0.001	0.001	0.002
Elevated cholesterol	Tertile 3	1.81 (1.23, 2.65)	1.83 (1.24, 2.71)	1.80 (1.21, 2.68)
Level	Tertile 2	1.17 (0.80, 1.71)	1.19 (0.81, 1.74)	1.18 (0.80, 1.73)
	Tertile 1	1	1	1
	Trend over tertiles, p	0.001	0.001	0.003
Low HDL-chol level	Tertile 3	1.66 (1.04, 2.64)	1.67 (1.04, 2.69)	1.40 (0.85, 2.29)
	Tertile 2	1.27 (0.81, 2.00	1.28 (0.81, 2.02)	1.16 (0.73, 1.85)
	Tertile 1	1	1	1
	Trend over tertiles, p	0.002	0.002	0.025
Type 2 diabetes	Tertile 3	1.14 (0.69, 1.88)	1.29 (0.76, 2.18)	1.20 (0.70, 1.97)
	Tertile 2	0.86 (0.52, 1.43)	0.96 (0.57, 1.63)	0.92 (0.54, 1.56)
	Tertile 1	1	1	1
	Trend over tertiles, p	0.575	0.273	0.566
Smoking	Tertile 3	1.25 (0.75, 2.10)	1.21(0.71, 2.06)	1.17 (0.67, 2.02)
	Tertile 2	0.95 (0.57, 1.56)	1.01 (0.60, 1.70)	0.99 (0.58, 1.68)
	Tertile 1	1	1	1
	Trend over tertiles, p	0.820	0.971	0.951
≥3 cardiovascular risk	Tertile 3	1.88 (1.30, 2.72)	1.88 (1.29, 2.74)	1.65 (1.12, 2.43)
Factors	Tertile 2	1.39 (0.97, 1.99)	1.41 (0.97, 2.04)	1.33 (0.91, 1.93)
	Tertile 1	1	1	1
	Trend over tertiles, p	0.001	0.001	0.008
Body mass index ≥25	Tertile 3	2.89 (1.86, 4.50)	2.86 (1.82, 4.50)	2.86 (1.82, 4.50)
kg/m^2^ (overweight-obesity)	Tertile 2	1.66 (1.08, 2.56)	1.68 (1.08, 2.60)	1.68 (1.08, 2.60)
	Tertile 1	1	1	1
	Trend over tertiles, p	0.001	0.001	0.001

^a^ Adjusted for physical activity (inactivity, insufficient activity, recommended activity) alcoholic beverages consumption per day (0, <2, >2), and family history of myocardial infarction.

^b^ Adjusted for body mass index (< 25 kg/m^2^, ≥ 25 kg/m^2^), in addition to the variables included in “a”.

^c^ Tertile 3: ≥ 5.35 mg/dL; tertile 2: 4.19 - 5.34 mg/dL; tertile 1: ≤ 4.18 mg/dL.

Regarding each of the factors which constitute the CR, in a multivariate model adjusted by physical activity, alcoholic beverages consumption, and family history of myocardial infarction we observed subjects in higher tertile of SUA had higher odds of suffering hypertension (OR = 1.86, 95% CI: 1.26-2.74), having high total cholesterol (OR = 1.83, 95% CI: 1.24-2.71) or low HDL-chol (OR = 1.67, 95% CI: 1.04-2.69), and higher odds of having three or more risk factors (OR = 1.88, 95% CI: 1.29-2.74) than those in lower tertile. Likewise, medium and high SUA levels were associated with higher odds of having overweight by 68.0% and by more than 100%, respectively (OR = 1.68, 95% CI: 1.08-2.60; OR = 2.86, 95% CI: 1.82-4.50). There was a linear trend of the OR for high CR, hypertension, high total cholesterol, low HDL-chol, and having three or more risk factors across increasing tertiles of uric acid (ρ < 0.05). We also observed that subjects located in the second tertile of SUA were more likely to belong to the age group of cardiovascular risk than those located in the first tertile (OR = 1.53, 95% CI: 1.04-2.24). After adjusting by BMI in addition to the above mentioned variables, our estimates of the association between high SUA levels and hypertension and between high SUA levels and the presence of three or more risk factors decreased, nonetheless, they kept their statistical significance (Table 4).

**Figure 2:**
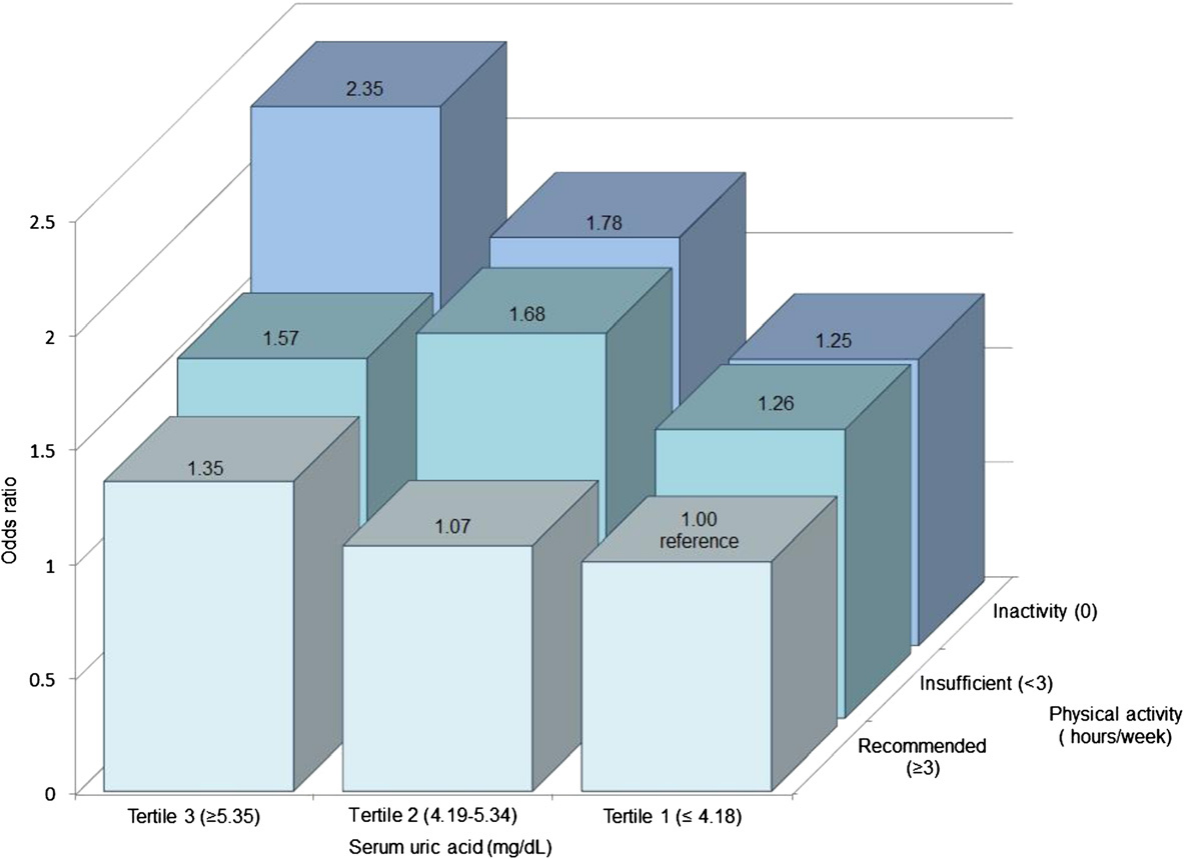
Odds ratios of cardiovascular risk by serum uric acid concentrations and physical activity levels in university workers of State of Mexico, 2004.

Further analysis of the interaction between physical activity and uric acid concentrations on CR revealed that the physically inactive individuals and with high levels of uric acid, had two-fold higher odds of presenting high 10-year CR in comparison to those physically active with low SUA levels (OR = 2.35, 95% CI:1.24 - 4.45) (Figure 2). An additional file shows the data from which was done the Figure 2 (see Additional file 1).

## Discussion

This study compared SUA levels among workers with and without 10-year CR at a public university in Mexico. Results show a positive association between SUA and CR in the crude analysis, but no association was found when the analysis was adjusted by physical activity, alcoholic beverages consumption, family history of myocardial infarction and body mass index. Among physically inactive individuals, SUA concentrations were positively associated with high CR.

Average concentration of uric acid among university workers (4.8 mg/dL) was found to be within the normal range, in accordance with information on other groups of asymptomatic middle-aged adults [25,26]. Moreover, consistent with previous studies, association between SUA and CR was obtained with uric acid levels considered to be normal to high range (5.3 mg/dL) [27,28]. In this respect, Hayden and Tyagi [8] point out that SUA values from the upper third of the physiologically normal range (> 4.0 mg/dL) have a harmful impact on the vascular endothelium leading to endothelial dysfunction through processes of oxidative stress.

The CR to ten years estimate was associated to higher SUA values, which suggests a close relationship between SUA and CR. In accordance with this result, recent studies describe an association between SUA and increased cardiovascular disease risk [29,30] as well as a higher prevalence of metabolic syndrome or its components [31,32]. Likewise, they point to the fact that in the population with a relatively low CR, uric acid is a weak predictor of cardiovascular morbidity and on the contrary, it could constitute a significant independent predictor of cardiovascular disease among individuals with high or very high CR [6].

Elevated SUA levels are commonly associated with CR factors such as hypertension [33], diabetes mellitus [34], hyperlipidemia and obesity [35]. In this study, exposure to high SUA levels was associated with a higher risk of displaying abnormal figures of blood pressure, HDL-chol, and total cholesterol with respect to non-exposure. Dawson and Walters [36] have proposed that the physiopathological mechanism through which it is possible to link uric acid with hyperlipidemia and atherosclerotic cardiovascular disease is the heightened activity of the enzyme xanthine oxidase. The superoxide anions generated by xanthine oxidase during the metabolism of purines can inactivate nitric oxide and thus lead to the formation of other oxidants, which under the effect of DNA and lipid oxidation contribute to the development of atherosclerosis.

In this study, excess weight was closely associated with both CR and SUA levels and constituted an important confounder of the relationship between these variables. High SUA levels were significantly associated with global CR in the crude analysis, and this association persisted after controlling for potential confounders; nevertheless, further adjustment for BMI caused a decrease in the strength of the association and loss of statistical significance. Recent studies show a positive association between obesity and uric acid [32,35], and some of them even posit that obesity is the main determinant of high SUA values in the general population [30]. It has also been observed that hyperuricemia precedes the development of obesity [35]. According to Sautin *et al.*[37], uric acid contributes to the development of obesity by giving rise to inflammatory and oxidative changes in adipocytes. Likewise, the relationship between SUA and hypertension was attenuated among individuals whose body weight was above normal, without losing statistical significance. Contrary to this result, in the study by Mellen *et al.*[11] the relationship between SUA and hypertension lost statistical significance among individuals with obesity, probably due to their higher average age (53.3 years) than individuals in this study (45.3 years), since recent scientific evidence sustains that the relationship between SUA and hypertension is attenuated with age [28].

On the other hand, our results suggest an interaction between uric acid levels and physical inactivity on the likelihood of developing cardiovascular disease in the next ten years. Physically inactive workers with high SUA levels were two fold more likely to have high CR in comparison with those physically active with low SUA levels. Concerning these relationships, previous studies reveal that physical activity is inversely related to both the cardiovascular morbidity [38] as the uric acid levels [39-41], and show there is a remarkable similarity between the features associated with elevated levels of uric acid and those associated with physical inactivity, such as endothelial dysfunction [8,42], inflammation [43], oxidative stress and insulin resistance. It has been proposed that the relationship between SUA and physical activity is mediated by the latter’s effect on insulin sensitivity [39,44], although it has also been posited that elevated uric acid levels may have a causal role in the pathogenesis of insulin resistance [45], since elevated levels of uric acid decrease the bioavailability of nitric oxide, and insulin requires endothelial nitric oxide to stimulate glucose uptake in skeletal muscle [46].

One of the limitations of this study is that the cohort from which participants were drawn included only university workers, so the results are not strictly applicable to the general population. Nevertheless, the study population represents a group with very diverse characteristics shared by the general population, such as the high prevalence of overweight-obesity (72.0%) and high physical inactivity (36.0%), which are similar to those reported by the National Survey of Health and Nutrition of Mexico 2006 [4]; hence, the results are potentially generalizable to the working population of urban areas in Mexico. Moreover, since we are dealing with a cross-sectional study, causality cannot be inferred between high SUA levels and CR. However, it is possible to infer that SUA is a good indicator of CR, especially among sedentary individuals.

## Conclusions

The results from this study in a middle-aged adult population in Mexico show that SUA levels are positively associated to high 10-year global CR among people with a sedentary lifestyle.

Even though the association between uric acid and risk of developing a cardiovascular disease has been observed among other ethnic groups, from results of this study it follows that in Mexico uric acid can be considered as an early marker of the risk of developing a cardiovascular event in the next ten years. This will prove very useful in terms of prevention strategies targeting these ailments.

## Competing interests

Authors had no commercial or other associations that might pose a conflict of interest in connection with the submitted article.

## Authors’ contributions

PC and BHP carried out the conception and design of the study, the analysis and the interpretation of the data and drafted the discussion. PC, ED, RV, GV and EC drafted the article, and GH approved the final version to be published. All authors reviewed and approved the final version of the manuscript.

## Pre-publication history

The pre-publication history for this paper can be accessed here:

http://www.biomedcentral.com/1471-2458/13/415/prepub

## Supplementary Material

Additional file 1 Odds ratios of cardiovascular risk by serum uric acid concentrations and physical activity levels in university workers of State of Mexico, 2004. Click here for file
