# Supplementary material for: Association between serum uric acid levels and cardiovascular risk among university workers from the State of Mexico: a nested case–control study
Source: BMC Public Health. 2013 May 1;13:415. doi: 10.1186/1471-2458-13-415 (PMC3648412; doi:10.1186/1471-2458-13-415)
Supplement: Additional file 1 — Odds ratios of cardiovascular risk by serum uric acid concentrations and physical activity levels in university workers of State of Mexico, 2004. [file 1471-2458-13-415-S1.docx]

**Appendix 1. Odds ratios of cardiovascular risk by serum uric acid concentrations and physical activity levels in university workers of State of Mexico, 2004**

| ***Factors*** | ***Odds ratio (95% CI)*** |
| --- | --- |
| Tertiles of uric acid (UA) concentrations ^§^ physical activity levels | |
| Tertile 3 of UA (n = 319) | 1.41 (0,60 3.31) |
| Tertile 2 of UA (n = 320) | 1.07 (0.60, 2.03) |
| Physical inactivity (n = 319) | 1.66 (0.91, 3.00) |
| Insufficient physical activity (n = 318) | 1.26 (0.64, 2.48) |
| Tertile 3 of UA ^§^ Physical inactivity (n = 100) | 2.35 (1.24, 4.45) |
| Tertile 2 of UA ^§^ Physical Inactivity (n = 101) | 1.78 (0.93, 3.42) |
| Tertile 1 of UA ^§^ Physical inactivity (n = 143) | 1.25 (0.68, 2.28) |
| Tertile 3 of UA ^§^ Insufficient physical activity (n = 99) | 1.57 (0.81, 3.05) |
| Tertile 2 of UA ^§^ Insufficient physical activity (n = 104) | 1.68 (0.88, 3.22) |
| Tertile 1 of UA ^§^ Insufficient physical activity (n = 91) | 1.26 (0.64, 2.48) |
| Tertile 3 of UA ^§^ Recommended physical activity (n = 119) | 1.35 (0.71, 2.56) |
| Tertile 2 of UA ^§^ Recommended physical activity (n = 115) | 1.07 (0.56, 2.03) |
| Tertile 1 of UA ^§^ Recommended physical activity (n = 85) | 1 (reference) |

^§^ Interaction
